# Bruton's tyrosine kinase (Btk) inhibitor tirabrutinib suppresses osteoclastic bone resorption

**DOI:** 10.1016/j.bonr.2019.100201

**Published:** 2019-03-15

**Authors:** Yuko Ariza, Masayuki Murata, Yoshiko Ueda, Toshio Yoshizawa

**Affiliations:** Ono Pharmaceutical Co., Ltd, Osaka, Japan

**Keywords:** Osteoclasts, RANKL, Bone erosion, Bruton's tyrosine kinase, Tec

## Abstract

Osteoclasts are responsible for bone erosion in osteoporosis and rheumatoid arthritis (RA). Both Btk and Tec kinases have essential functions in osteoclast differentiation. Tirabrutinib is a highly potent and dual oral Btk/Tec inhibitor with an IC_50_ in the nmol/L range and significantly inhibits the M-CSF and RANKL-driven osteoclast differentiation. It was hypothesized that the *in vitro* activity of tirabrutinib could be demonstrated in mice bone resorption model. The RANKL model studies show that tirabrutinib significantly suppressed bone loss with the inhibition of serum TRAPCP5b and urinary CTX-1. Bone Mineral Density (BMD) loss in tirabrutinib-treated mice was 55% (*P* < .05), 87% (*P* < .001) and 88% (*P* < .001) for the 3, 10 and 30 mg/kg dose groups respectively.

Btk and Tec are required for osteoclast differentiation and activation based on the genetic evidence obtained from Btk and Tec double deficient mice. Tirabrutinib may be a novel therapeutic target for bone diseases, such as osteoporosis and RA.

## Highlights

• Btk and Tec are activated by RANKL and indispensable for osteoclastogenesis.
• Tirabrutinib is a highly potent and dual oral Btk/Tec inhibitor.
• Osteoclast differentiation is activated by RANKL, M-CSF and ITAM.
• Tirabrutinib inhibited RANKL-induced osteolyrtic bone disease.

## 1 Introduction

Osteoporosis is a disease of increased bone fragility, leading to a higher risk of breaks or fractures (Ramírez et al., 2018). A minor bump or fall can be enough to cause a break in someone with osteoporosis. The primary objective of treating patients with or who are at risk for osteoporosis is to reduce the likelihood of new fractures. Bisphosphonates and teriparatide reduce the incidence of non-vertebral fracture (Yusuf et al., 2018), and both alendronate and risedronate decrease the incidence of hip fracture in postmenopausal women with osteoporosis by about 50% (Liberman et al., 1995; McClung et al., 2001). In general these therapies are well tolerated. However, the effectiveness of treatment is limited by poor adherence to therapy, as most patients discontinue treatment before the end of the first year (Pavone et al., 2017).

Receptor activator of nuclear factor-κB (RANK) is a member of the tumor necrosis factor family expressed by osteoclasts and their precursors (Hofbauer et al., 2000). The interaction of RANK with its ligand (RANKL) leads to the recruitment of adaptor proteins, TRAF6 and the rapid activation of MAPKs, NF-κB, and activator protein-1 (AP-1) (Kobayashi et al., 2001; Teitelbaum and Ross, 2003). Osteoprotegerin (OPG) is the natural inhibitor of RANKL. Mice that are deficient in OPG exhibit osteoporosis, whereas over-expression of OPG in mice results in reduced numbers of osteoclasts and high bone mass (Bucay et al., 1998). Many of these molecules that are involved in RANK signaling are also components of other signaling pathways. Thus, the manner in which common molecules are regulated to ensure the specific signal transduction required for osteoclast formation remains unclear.

Bruton's tyrosine kinase (Btk) is a member of TEC family (Btk, Bmx, Itk, Rlk and Tec) kinase (Qiu and Kung, 2000) that is broadly expressed in cells of several hemopoietic lineages, but not in T-cells, NK cells and plasma cells (Tomlinson et al., 2004). Genetic evidence supports a critical role for Btk in multiple hematopoietic signaling pathways including the B-cell receptor (BCR), several cytokine receptors and a potential novel role in heterotrimeric G-protein-associated receptors related to B-cell migration and adhesion, such as the CXCR4 and CXCR5 chemokine receptors and adhesion molecules, integrins (Musilova and Mraz, 2015; de Gorter et al., 2007). Mutations within Btk result in arrested B-cell development leading to severe X-linked agammaglobulinemia (XLA) (Moreau et al., 2007). These observations have indicated that Btk is a functional as opposed to a genetically-defined therapeutic target and has led to the development of many small molecule Btk inhibitors for the treatment of B-cell malignancies (Hendriks et al., 2014) and autoimmune disorders (Di Paolo et al., 2011). X-linked immunodeficiency (Xid) – a similar, although less severe syndrome in mice – is also the product of a *Btk* point mutation (Tarakhovsky, 1997). Interestingly, bone marrow cells derived from Xid mice undergo retarded osteoclastogenesis (Lee et al., 2008), but there is no obvious bone phenotype *in vivo*, suggesting compensation by other family members. Indeed, double deletion of Btk and Tec leads to severe osteopetrosis due to cell-autonomous blockade of osteoclast differentiation *in vitro* an *in vivo* (Shinohara et al., 2008). RANKL-induced phosphorylation of Btk and Tec complex with the SH2-containing leukocyte protein (SLP) family adaptor, B-cell linker protein (BLNK) (Lee et al., 2008) and therefore, it may be the molecular switch integrating RANK and ITAM signals.

Tirabrutinib is a covalent type inhibitor with comparable efficacy to ibrutinib in the treatment of B-cell malignancies (Walter et al., 2016) and has greater selectivity for Btk (*in vitro* IC_50_, 2 nmol/L) and Tec (*in vitro* IC_50_, 5 nmol/L) than other kinases, including Lck, Fyn, Lyn and Itk (KINOMEscan platform: 442 kinases) (Yasuhiro et al., 2017). Tirabrutinib inhibits cell proliferation in some malignant B-cell lines but does not inhibit the activation of T-lymphocytes from human PBMCs (Kozaki et al., 2018).

Herein, we extended our studies and evaluated the effect of tirabrutinib on a murine bone resorption model. The data indicate that tirabrutinib could be effective in bone diseases.

## 2 Materials and methods

### 2.1 Animal used

Seven-week-old female of C57BL/6NCrlCrlj mice (Charles River Laboratories Japan, Inc.) were used. All mice were allowed free access to pelleted CRF-1 diet (Oriental Yeast Co., Ltd.) and tap water. The present study was conducted in compliance with the “Guidance for Animal Experiments,” the “Ethical Standards for Experiments using Human Tissues,” and the “Standards for Safety Management of Pathogens” established by Ono Pharmaceutical Co., Ltd.

### 2.2 Reagents

Tirabrutinib, ibrutinib, fostamatinib, tofacitinib were obtained from the Medicinal Chemistry Research Laboratories, Ono Pharmaceutical Co., Ltd. (Osaka, Japan). Anti-mouse RANKL monoclonal neutralizing antibody (hereinafter referred to as anti-RANKL antibody) was from Oriental Yeast Co., Ltd. p38 inhibitor was used as a positive control (Tao et al., 2011).

### 2.3 Preparation and differentiation of human osteoclast precursor cell

Human osteoclast precursor cells (Lonza) were cultured with 33 ng/mL M-CSF and 66 ng/mL RANKL for 7 days according to the manufacturer's protocol (Lonza). The Acid Phosphatase, Leukocyte (TRAP) Kit (Sigma-Aldrich) was used for tartrate-resistant acidic phosphatase (TRAP) staining. Staining was performed in accordance with instructions, and TRAP-positive multinucleated (≥3) cells were counted as osteoclasts under a microscope. Stained cells were photographed using an HS All-in-One Fluorescence Microscope and BZ-II Image Analysis Application to obtain image data.

### 2.4 Cytotoxicity assay

The CellTiter-Glo Luminescent Cell Viability Assay was used. Luminescence signals (relative luminescence unit, RLU) in proportion to the amount of intracellular ATP were measured using a microplate reader (SpectraMax M5e, Molecular Devices, Inc.) in accordance with instructions.

### 2.5 Western blot analysis

Total lysates of human osteoclast precursor cells were prepared from the lysis buffer (Cell Signaling Technology). Total lysates were loaded on 4–12% SDS-PAGE (Bio-Rad), and then western blot analysis was performed using antibodies, pBtk Y223 (Novus Biologicals), pLyn Y396 (Gene Tex), Btk, Lyn, pGab2 Y452, Gab2, pPLCγ2 Y759, PLCγ2, pBLNK, BLNK, NFATc1 (Cell Signaling Technology).

### 2.6 RANKL-induced bone loss

Female C57BL/6NCrlCrlj mice were injected intraperitoneally with 20 μg/body of RANKL (Oriental Yeast Co., Ltd.) three times at 24 h intervals from day 0 to day 2. Tirabrutinib, tofacitinib and fostamatinib were administered orally twice daily for a total of 5 administrations from day 0 to day 2. Anti-RANKL antibody was administered subcutaneously in the dorsocervical portion at a volume of 10 mL/kg using a 26-gauge tuberculin syringe for twice on day −7 and day −4 prior to the first induction day.

### 2.7 Structural analysis of trabecular bone (μCT)

The distal end of the right femur was structurally analyzed using the μCT40 cone-beam micro-CT scanner (Scanco Medical), the attached AlfaStation DS10 workstation (COMPAQ), and the Image Language analytical software program (IPL, ver.3.1). Femurs were placed in a measuring vessel, and 34 two-dimensional images at 0.012 μm slice thickness were taken in the proximal direction from a position 0.5 mm from the femoral distal growth plate using an X-ray source. The volume of interest (VOI) selected was the trabecular bone region of the distal femur. With a threshold value of 182 for trabecular bone and marrow components, three-dimensional images were produced to obtain various parameters of bone microstructure. Likewise, 58 images at 0.012 μm slice thickness were taken in the proximal direction from a position 0.5 mm from the femoral distal growth plate to obtain three-dimensional images of the whole bone. BV/TV: Bone volume/Tissue volume (%), Conn.Dens: Connective Density, SMI: Structure Model Index (structure evaluated as a score from 0 to 3, where, plate-like: 0, rod-like: 3), Tb.N: Trabecular Number (/mm), Tb.Th: Trabecular Thickness (μm), Tb.Sp: Trabecular Separation (mm).

### 2.8 Determination of bone mineral density (pQCT)

After removing muscles and other soft tissues, femurs were placed in a measuring vessel, and bone mineral density of the femoral distal metaphysic (0.6 mm proximal from the femoral distal growth plate) was determined using XCT Research SA+ (software version 5.50, Stratec Medizintechnik GmbH). Trabecular bone mineral density was calculated in contour mode 2 and peel mode 20 with a ratio to whole bone cross-sectional area of 35%.

### 2.9 Measurement of TRACP5b

At 1.5 h following induction on day 2, approximately 500 μL of blood was drawn from the inferior vena cava under isoflurane anesthesia and mice were subsequently exsanguinated. Collected blood was then transferred to a 1.5 mL sampling tube and allowed to stand at ambient temperature for 30 min or more, after which the blood was centrifuged at 4 °C, 12,000 rpm for 10 min and supernatant, as serum, was transferred to a new tube and stored at −80 °C until measurement. Mouse TRACP5b ELISA (ImmunoDiagnostics) was used for TRACP5b.

### 2.10 Measurement of CTx

After RANKL induction, urine was collected over time by compressing the lower abdomen of each immobilized mouse and stored at −20 °C until measurement. RatLaps ELISA (ImmunoDiagnostics) was used. In addition, urinary creatinine (CRE) concentrations in the same samples were measured using Creatinine Microplate Assay (Oxford Biomedical Research). Urinary CTx concentrations were calculated using the equation below:

$$\text{Urinary}\;\mathrm{CTx}\;\text{concentration}\;\left(\mathrm{\mu g}/\text{mmol}\;\mathrm{CRE}\right)=\mathrm{CTx}\;\text{value}\;\left(\mathrm{\mu g}/L\right)/\left\{\mathrm{CRE}\;\text{concentrations}\;\left(\mathrm{mg}/\mathrm{dL}\right)\times 0.088\right\}$$

### 2.11 Statistics

SAS 9.1.3 (EXSUS Ver. 7.7.1, SAS Institute Japan Ltd. [CAC EXICARE Corporation]) was used for analysis of animal data. Data are expressed as the mean ± standard error. All statistical tests were two-sided with a significance level of 5%.

## 3 Results

### 3.1 BTK plays a role on osteoclasts differentiation

**Fig. 1:**
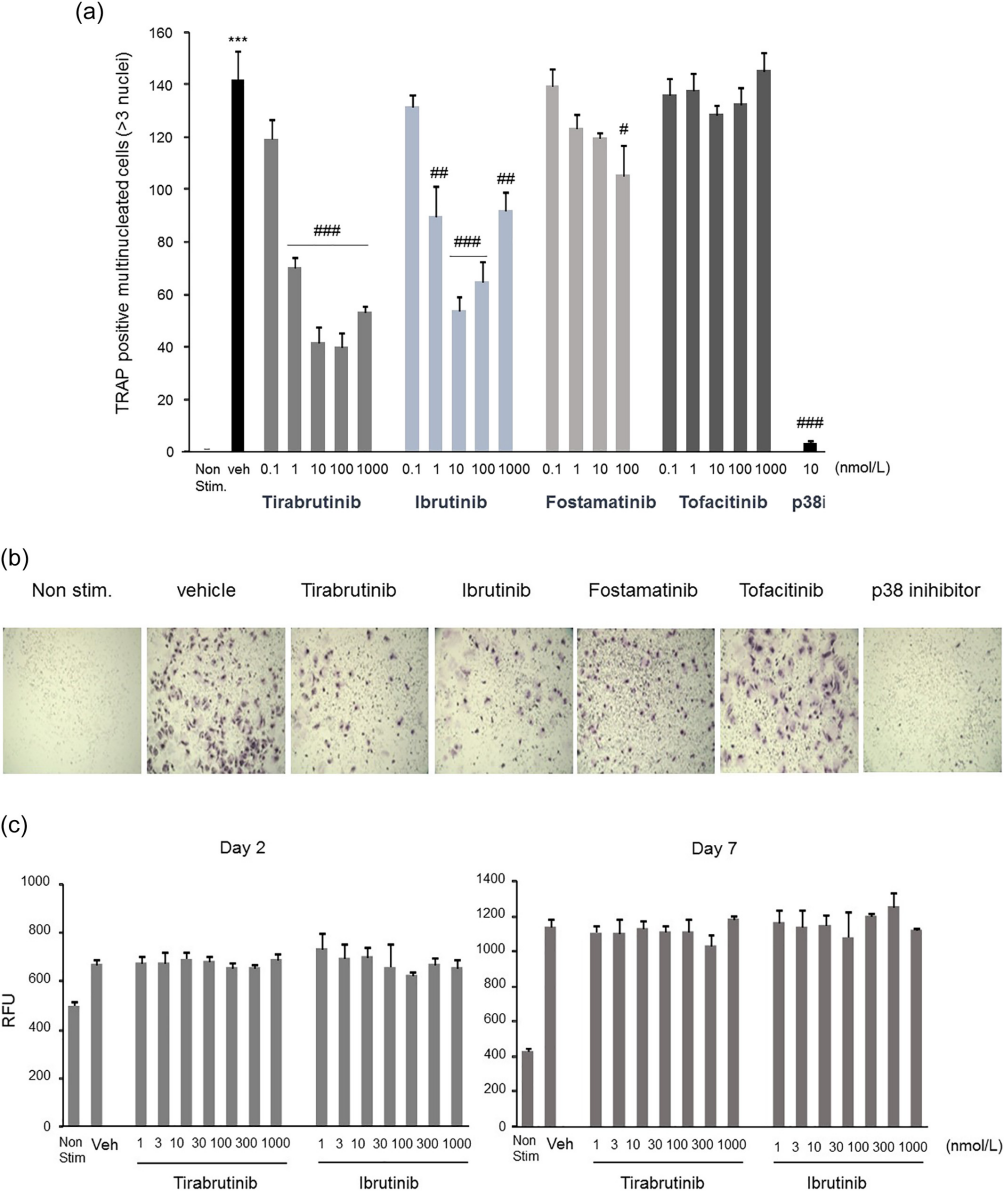
Effects of various inhibitors on mature osteoclast differentiation of human osteoclast precursor cells. Human osteoclast precursor cells were prepared in media containing M-CSF and RANKL. The cells were treated with tirabrutinib, ibrutinib, fostamatinib and tofacitinib at concentrations from 0.1 to 1000 nmol/L for 7 days. Osteoclast differentiation was evaluated by TRAP staining and counting of TRAP+ cells containing at least three nuclei on day 7. (a) Inhibitory effect is expressed as the mean ± standard error (*n* = 3 samples per group). ***: *p* < .001 *vs* Non-stimulation group. #, ##, ###: *p* < .05, *p* < .01, *p* < .001 *vs* group with vehicle. (b) Cells after TRAP staining were photographed under a light microscope. All compounds were treated at 100 nmol/L, except for positive control, p38 inhibitor at 10 nmol/L. (c) Cytotoxic effects of tirabrutinib and ibrutinib on human osteoclast precursor cells. After completion of cultivation, RLU was measured. Cytotoxicity is expressed as the mean ± standard error (*n* = 3 samples per group).

Previous studies demonstrated that ibrutinib, Btk inhibitor, suppresses osteoclastogenesis in a mouse collagen-induced arthritis model (Shinohara et al., 2014). To explore the mechanism of tirabrutinib on osteoclast regulation, we first evaluated osteoclast differentiation in primary human osteoclast precursor cells. Human osteoclast precursor cells were prepared and stimulated with RANKL and M-CSF, and then treated with Btk and other kinase inhibitors (Syk, Jak and p38) for 7 days. The effect of Btk inhibitors (tirabrutinib, ibrutinib), Syk inhibitor (fostamatinib) and Jak inhibitor (tofacitinib) on osteoclast differentiation was evaluated. Tirabrutinib inhibited osteoclast differentiation and the IC_50_ and 95% confidence intervals were 1.35 nmol/L and 1.03 to 1.78 nmol/L, respectively. Ibrutinib also inhibited osteoclast differentiation, however, unlike tirabrutinib, the effect was lower at 100 and 1000 nmol/L (Fig. 1a, b). Given a cytotoxic effect was observed at 300 nmol/L, fostamatinib showed minimum inhibition at 100 nmol/L as the maximum concentration for subsequent incubation for 7 days. Tofacitinib exhibited no inhibitory effect. The cytotoxic effects of tirabrutinib and ibrutinib on human osteoclast precursor cells were evaluated. Both compounds exhibited no cytotoxic effects at concentrations ranging from 1 to 1000 nmol/L until Day 7 (Fig. 1c).

### 3.2 Differential signaling of tirabrutinib and ibrutinib for osteoclast regulation

**Fig. 2:**
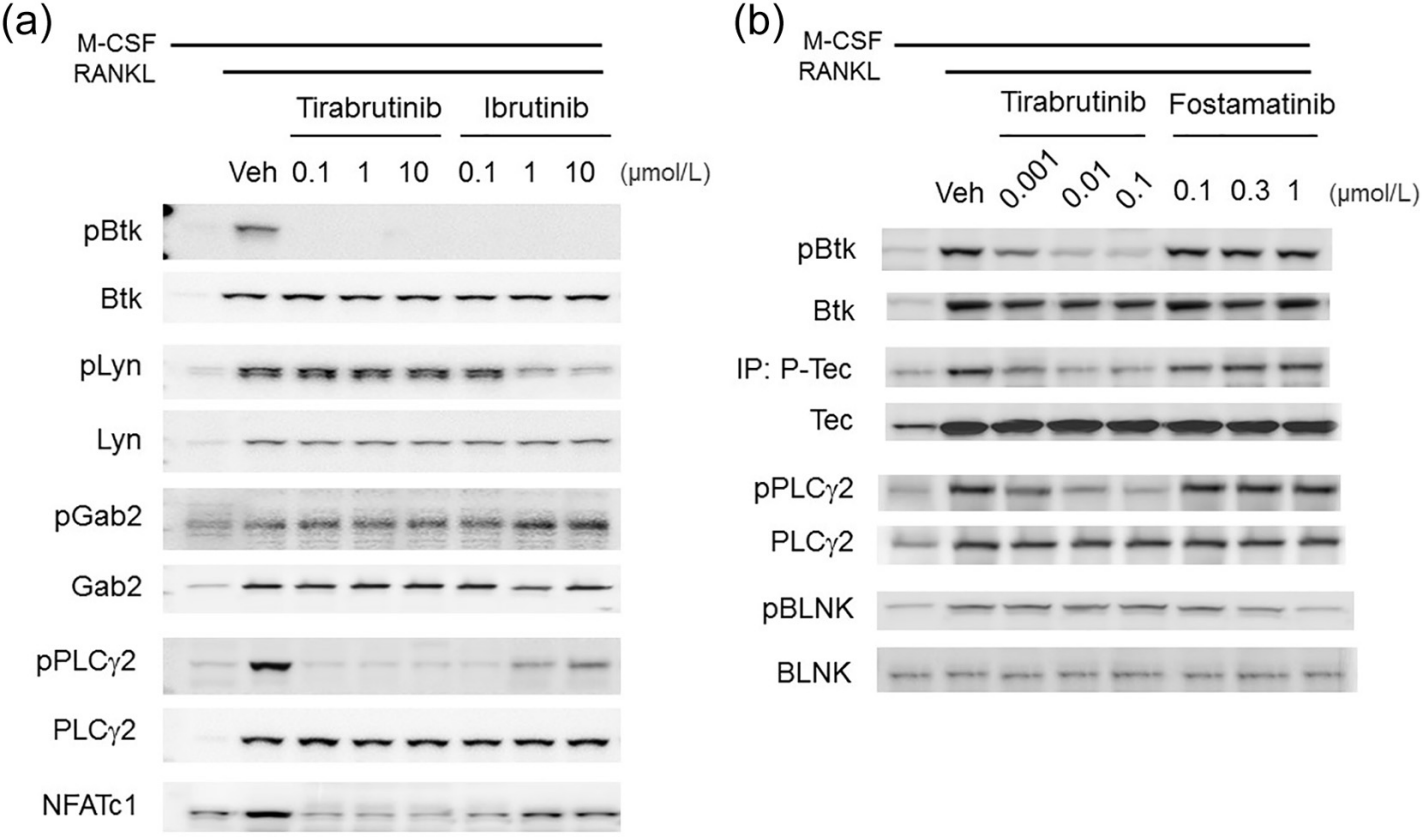
Btk mediates Osteoclast signaling pathway. (a, b) Primary human osteoclast precursors were stimulated with M-CSF and RANKL for 48 h. Intracellular phosphorylated Btk (pBtk), total Btk protein (Btk), phosphorylated Lyn (pLyn), total Lyn protein (Lyn), phosphorylated Gab2 (pGab2), total Gab2 protein (Gab2), phosphorylated PLCγ2 (pPLCγ2), total PLCγ2 protein (PLCγ2), phosphorylated BLNK, total BLNK and NFATc1 were detected by Western blot analysis. (b) After immunoprecipitation with an anti-Tec antibody, phosphorylated tyrosine (pTyr) and Tec protein (Tec) were detected by Western blot analysis.

The unexpected result of ibrutinib on osteoclast differentiation led us to focus on RANKL signaling. (Fig. 2a) shows the western blot analysis of osteoclasts in the presence of M-CSF and RANKL for 48 h. The Btk phosphorylation (pBtk) was completely inhibited after treatment with both tirabrutinib and ibrutinib. In contrast, Lyn phosphorylation (pLyn) was only inhibited by ibrutinib at higher concentrations, 1 and 10 μmol/L. Since Btk is downstream of Lyn, the inhibition effect of Lyn by ibrutinib was not due to the higher potency of Btk inhibition. Surprisingly, bands with PLCγ2 phosphorylation and NFATc1 were observed at the higher concentrations of ibrutinib despite both are downstream of Btk. It has been reported that both tirabrutinib and ibrutinib have inhibitory activity on Tec kinase (Walter et al., 2016; Honigberg et al., 2010). The biochemical activity against recombinant human Tec is 5 nmol/L for tirabrutinib and 77.8 nmol/L for ibrutinib (about 16-fold less potent than tirabrutinib). Tirabrutinib inhibited both Btk and Tec phosphorylation but did not influence BLNK phosphorylation. While fostamatinib has no activity against Btk/Tec but did influence BLNK phosphorylation, shown in (Fig. 2b). These data suggest that tirabrutinib suppresses RANKL signaling *via* an inhibition of Btk/Tec.

### 3.3 Effects of tirabrutinib on mechanical properties of bone

**Fig. 3:**
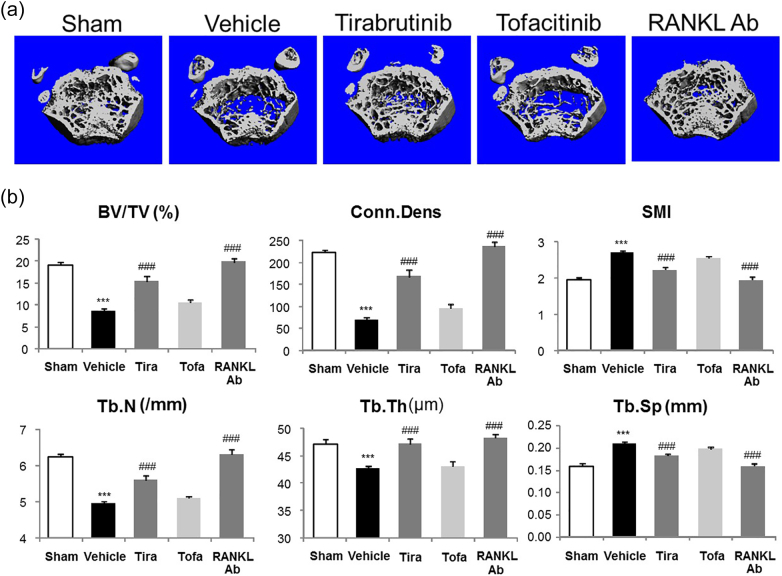
Effects of tirabrutinib on mechanical properties of bone. Bone loss was induced in C57BL/6N mice by intraperitoneally administering RANKL every 24 h for a total of 3 administrations. Tirabrutinib or tofacitinib at each dose was administered orally twice daily for a total of 5 administrations from day 0 to day 2. Anti-RANKL antibody was subcutaneously administered twice in total on day −7 and day −4. At 1.5 h following the final induction, blood was collected and the femur was excised. (a) For the distal end of the femur, the trabecular structure was analyzed by μCT to obtain 3-dimensional images. (b) Data are expressed as the mean ± standard error for 10 animals in each group.***: *p* < .001 *vs* Sham group. ###: *p* < .001 *vs* group with vehicle.

We then evaluated the effect of tirabrutinib, better selectivity for Btk, on RANKL-induced bone loss model. μCT and trabecular bone mineral density (BMD) were performed. C57BL/6N mice were injected intraperitoneally with RANKL three times at 24 h intervals. Treatments were initiated at the first day of induction and continued twice daily until the mice were sacrificed. Tirabrutinib treated mice had similar cortical architecture to the sham (pseudo treatment mice) and RANKL-Ab treated mice. In contrast, tofacitinib treated mice had worse cortical bone properties, like vehicle treated mice (Fig. 3a). Tirabrutinib also significantly influence trabecular bone microstructural parameters such as BV/TV, Conn. Dens, SMI, Tb.N, Tb.Th, Tb.Sp (Fig. 3b).

### 3.4 Role of BCR signaling on RANKL-induced bone loss

**Fig. 4:**
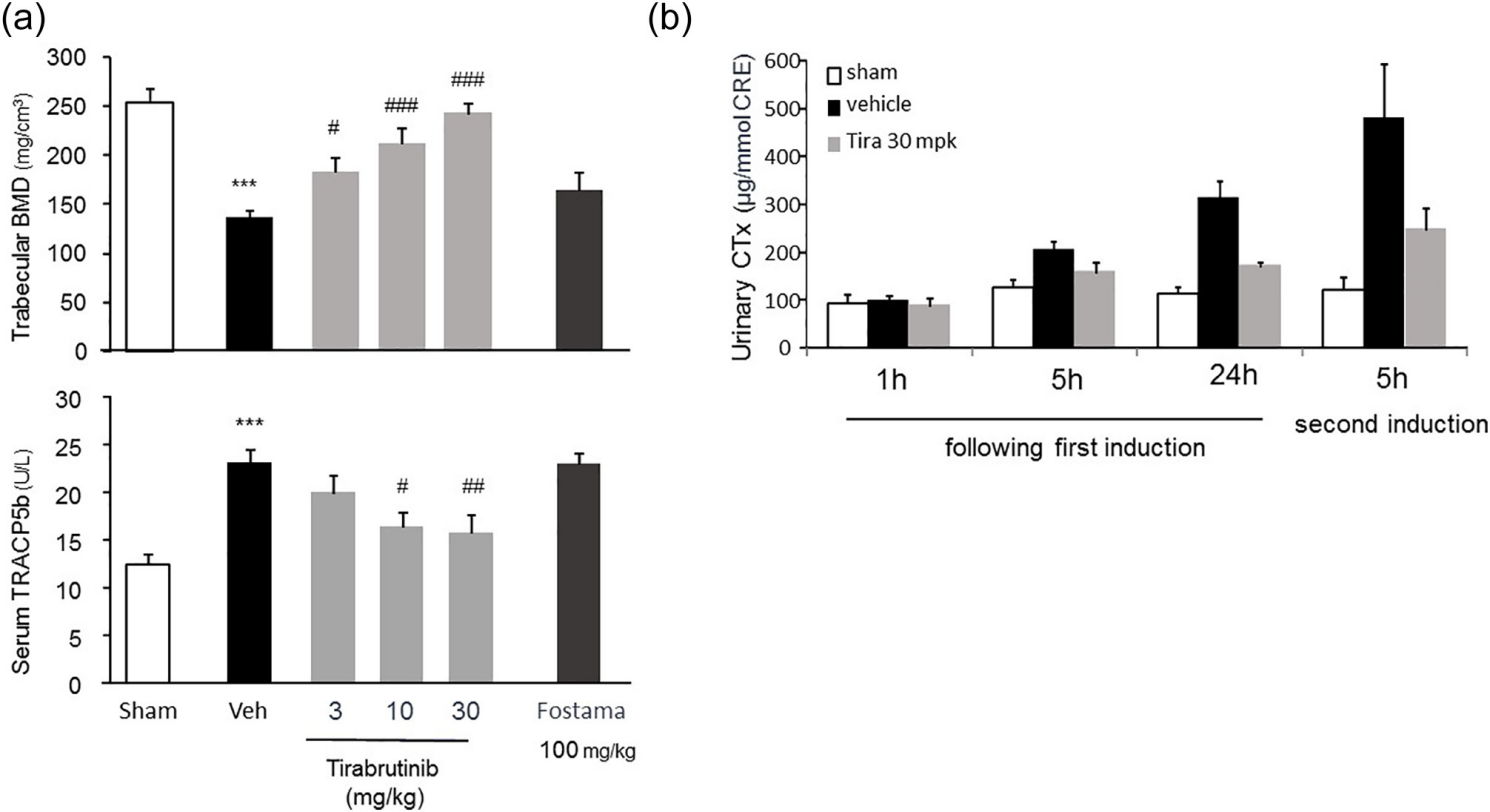
Effect of reproducibility of tirabrutinib and comparison with another BCR signaling modulator on RANKL-induced bone loss. Tirabrutinib or fostamatinib at each dose was administered orally twice a day (5 times in total). At 1.5 h following the final induction, blood was collected and the femur was excised. (a) Bone mineral density of the distal end of the femur was measured by pQCT. Serum TRACP5b concentrations were measured by ELISA. Data are expressed as the mean ± standard error for 10 animals in each group. The *t*-test was used to compare the sham and vehicle group. Dunnett's test was used to compare the vehicle and tirabrutinib groups. ***: *p* < .001 *vs* Sham group. #, ##, ###: *p* < .05, *p* < .01, *p* < .001 *vs* group with vehicle. (b) Tirabrutinib at a dose of 30 mg/kg was administered orally twice daily (5 times in total). At 1, 5, and 24 h following the first induction and at 5 h following the second induction, urine was collected by compressing the lower abdomen of each immobilized mouse. Urinary CTx concentrations were measured by ELISA. Data are expressed as the mean ± standard error for 10 animals in each group.

**Fig. 5:**
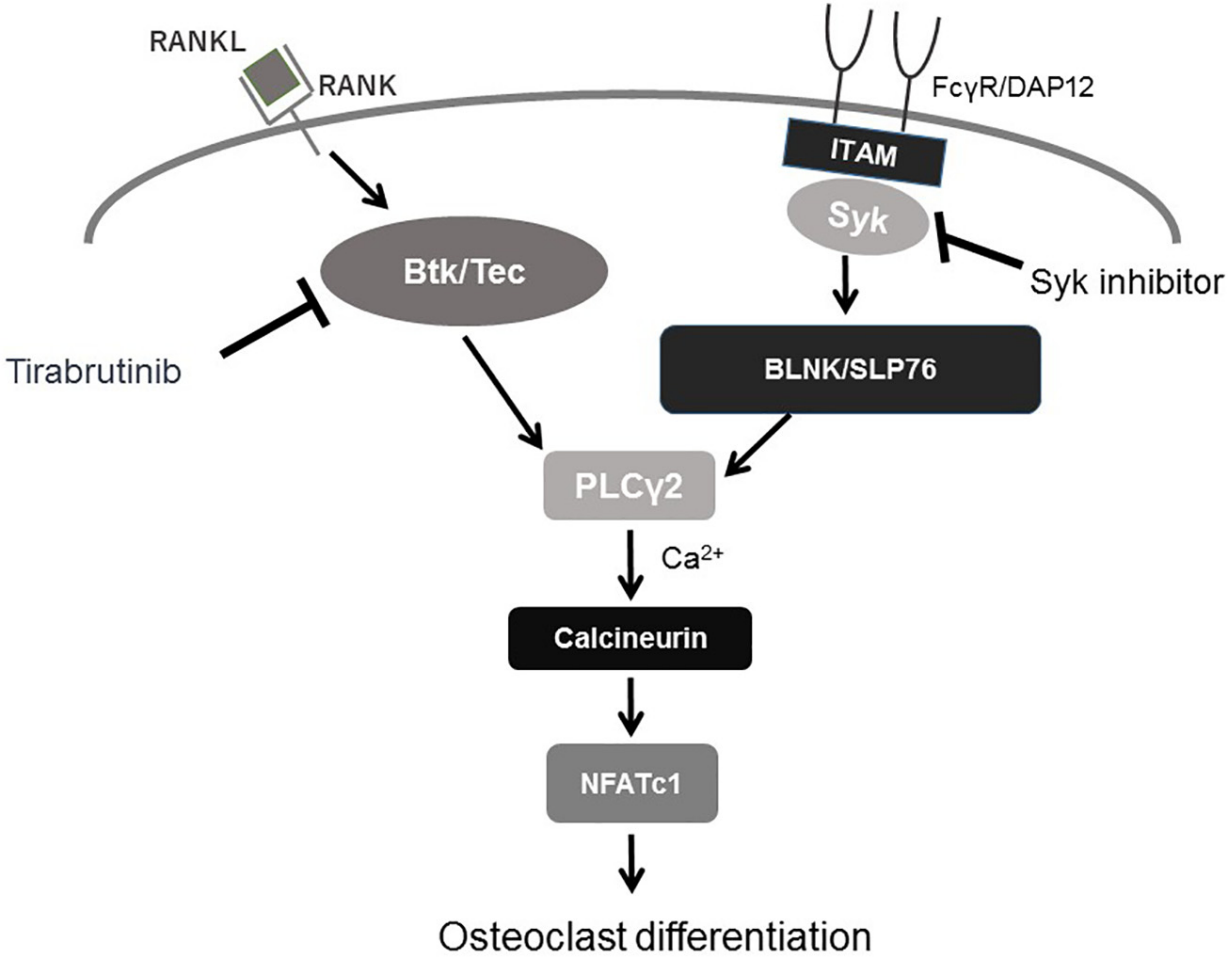
RANKL-RANK signaling pathway *via* Btk in osteoclast differentiation Tec family kinases are activated by RANK and bind to scaffolding proteins (BLNK or SLP76) activated by ITAM signaling, thereby forming a complex that cooperates to activate PLCγ2. PLCγ2 in turn stimulates calcium signaling, which is required for activation of NFATc1, a transcription factor essential for osteoclast differentiation. Our data suggest that ITAM signaling may be bypassed by RANK-RANKL signaling *via* Btk/Tec.

To investigate how BCR signaling involved in the observed bone deficiency, we tested whether Syk regulated downstream effectors of RANKL-mediated signaling. The effect of tirabrutinib and a potency comparison with fostamatinib are shown in (Fig. 4a). Tirabrutinib dose-dependently inhibited RANKL-induced BMD reduction and serum TRACP5b elevation. The inhibition rate was 59.5, 76.2, and 86.1% for BMD and 42.7, 58.6, and 74.8% for TRACP5b at the dose of 3, 10, and 30 mg/kg, respectively. In contrast, the inhibition rate exhibited by 100 mg/kg of fostamatinib was 23.7% for BMD reduction and 1.0% for TRACP5b elevation, respectively. These results demonstrated that Btk and Syk have distinct functions in BCR signaling after RANKL stimulation. Because Btk plays a pivotal role in osteoclast differentiation and activation, our data suggest that ITAM signaling may be bypassed by RANK-RANKL signaling *via* Btk/Tec (Fig. 5). Evaluation of urinary CTx concentration over time following RANKL induction is shown in (Fig. 4b). The CTx concentrations in the vehicle treated mice at 5 and 24 h after the first induction and 5 h after the second induction increased 1.6-, 2.8-, and 4.0-folds compared to the sham, showing increased CTx production over time. The inhibition rate exhibited by 30 mg/kg of tirabrutinib was 59.5, 71.6, and 64.8% at the respective time points.

## 4 Discussion

RANKL-RANK system in osteoclast biology has revealed critical signaling pathways and molecules regulating osteoclast differentiation. Osteoclast precursor cells that express RANK recognize RANKL and differentiate into osteoclasts in the presence of M-CSF. M-CSF induces the proliferation of pre-osteoclasts cells, sustains their survival and stimulates the expression of RANK (Scheven et al., 1998). RANKL expression by bone stromal cells, such as osteoblasts, synovial fibroblasts and T-cells increased adjacent to sites of pathological bone loss. The relative hierarchy of RANKL-RANK signaling cascades in osteoclast precursor cells has not been defined.

We used p38 MAPK inhibitor as a positive control for RANKL-induced osteoclastogenesis *in vitro*, given the evidence that attenuation of phosphorylation status of p38 leads to the inhibition of transcription factor, NFATc1 at the protein level (Pivetta et al., 2011; Choi et al., 2013). We observed the compound of p38 strongly inhibited osteoclast differentiation. Next, we examined how other kinase signaling pathways mediate RANKL-induced osteoclast differentiation. In this study, we found that Btk inhibitor, tirabrutinib inhibited osteoclast differentiation in a concentration-dependent manner. However, the efficacy of ibrutinib shows bell-shaped concentration response curves. One interpretation of these data is that ibrutinib inhibits off-targets for osteoclast differentiation at higher concentration. It often remains a significant challenge in use of kinase inhibitors with broad-spectrum for identifying the functional effect of off-target kinase. Previous studies demonstrated that Lyn deficiency enhances osteoclastgenesis *in vitro* and *in vivo* (Kim et al., 2009), indicating that Lyn is a negative regulation of osteoclastgenesis by RANKL. Our studies showed that the higher concentration of ibrutinib regulates Lyn activity and attenuates the inhibition of PLCγ2. The result is consistent with the fact that Lyn inhibits osteoclast differentiation by interfering with PLCγ-mediated Ca^2+^ signaling (Yoon et al., 2009).

Subsequent investigations sought to determine whether Btk, Syk or Jak regulates RANKL-induced bone loss model which is known as a model of short-time osteoclast differentiation and bone resorption caused by inducing bone resorption by mature osteoclasts (Tomimori et al., 2009). Tirabrutinib inhibited bone resorption as potent as anti-RANKL antibody. On the other hand, the bone resorption inhibitory effect of fostamatinib was found to be less effective at approximately 20%, as with the effect on *in vitro* human osteoclast differentiation, suggesting that Syk may not be located upstream of Btk in the RANKL-RANK signal transduction pathway. As expected, tofacitinib did not inhibit the aggravation of trabecular BMD and serum TRACP5b. It has been reported that tofacitinib, a pan-Jak inhibitor, and baricitibib, a Jak1/2 inhibitor, had no effect on RANKL-induced osteoclast differentiation (LaBranche et al., 2012; Murakami et al., 2017).

In conclusion, Btk and Tec are required for osteoclast differentiation and activation based on the genetic evidence obtained from Btk and Tec double deficient mice. This study demonstrates that dual Btk and Tec inhibitor, tirabrutinib exhibits potential therapeutic value for bone diseases, such as osteoporosis and rheumatoid arthritis.
